# Integrated histopathology of the human pancreas throughout stages of type 1 diabetes progression

**DOI:** 10.21203/rs.3.rs-6673858/v1

**Published:** 2025-06-10

**Authors:** Dirk Homann, Verena van der Heide, Sara McArdle, Michael Nelson, Karen Cerosaletti, Sacha Gnjatic, Zbigniew Mikulski, Amanda Posgai, Irina Kusmartseva, Mark Atkinson

**Affiliations:** University of Miami; Icahn School of Medicine at Mount Sinai; La Jolla Institute for Immunology; University of Wisconsin-Madison; Benaroya Research institute; Icahn School of Medicine at Mount Sinai; La Jolla Institute for Immunology; Department of Pathology, Immunology, and Laboratory Medicine, Diabetes Institute, College of Medicine, University of Florida, Gainesville, FL, USA; University of Florida; University of Florida

## Abstract

Type 1 diabetes (T1D) is a progressive autoimmune condition that culminates in loss of insulin-producing beta cells. Pancreatic histopathology provides essential insights into disease initiation and progression yet an integrated perspective onto *in situ* pathogenic processes is lacking. Here, we combined multiplexed immunostaining, high-magnification whole-slide imaging, digital pathology, and semi-automated image analyses to interrogate pancreatic tail and head sections across T1D stages, including at-risk and at-onset cases. Deconvolution of architectural features, endocrine cell composition, immune cell burden, and spatial relations of ~ 25,000 islets effectively contextualizes established and novel pancreatic hallmarks in health and T1D disease. Our results reveal a spatially homogenous and islet size-contingent architectural organization of the endocrine pancreas, a notable coordination of organ-wide pathogenic processes, and multiple histopathological correlates that foreshadow distinctive T1D histopathology already at the preclinical stage. Altogether, we propose a revised natural history of T1D with implications for further histopathological investigations and considerations of pathogenetic modalities.

## INTRODUCTION

Type 1 diabetes (T1D) is a chronic autoimmune disease characterized by the destruction of insulin-producing beta cells in pancreatic islets^1,2^. An interplay of genetic susceptibility^3^, environmental factors^4^, and immune dysregulation^5^ promotes multiple pathological alterations including pancreatic inflammation, islet-associated immune cell infiltration (insulitis), exocrine abnormalities, and systemic complications due to overt hyperglycemia^6–8^. Clinically, T1D progresses through three major stages: emergence of ≥ 2 T1D-associated islet autoantibodies but maintenance of normoglycemia (stage 1); additional development of dysglycemia (stage 2); and eventual clinical disease (stage 3)^9^.

Early histological interrogations of the pancreas established T1D as an autoimmune disease^10^ and defined pathological hallmarks^11^, yet extensive *in situ* studies have mostly been limited to the past ~ 15 years following the establishment of specialized tissue repositories such as the Network for Pancreatic Organ donors with Diabetes (nPOD) program^12^. Nevertheless, estimates suggest that < 700 T1D donor pancreata are available for research globally^13^, a limited resource that is further compounded by substantial heterogeneity at the level of organ donors, pancreas anatomy (*e.g.*, organ region, lobular insulitis), islet properties (*e.g.*, architecture, endocrine composition, association with immune cells), and altered endocrine function beyond beta cells (*e.g.*, alpha cells^14,15^). Pancreata from donors at high risk (stage 1/2, elevated HLA class-II risk) and at-onset of symptomatic T1D, critical for pathogenesis studies, are exceptionally scarce.

In order to contend with these challenges, emerging investigative techniques such as whole-slide imaging^11,16–27^, high-dimensional tissue analysis^26,28–34^, and three-dimensional (3D) visualization of immuno-stained thick pancreas slices^35,36^ have been deployed in conjunction with tailored digital analysis software. However, to date no standardized data analytics platform dedicated to pancreatic histopathology is readily accessible. Commercial platforms featuring “pancreas modules” and machine learning algorithms have aided the identification of pancreas and islet features^19,21,26,31,37–39^, but their proprietary nature restricts customization.

To our knowledge, a broad integration of the complementary strands of histopathological T1D pancreas investigation and digital pathology modalities^12,40^ has not yet been performed. To this extent, we combined higher dimensional multiplex brightfield immunohistochemistry (IHC), whole-slide image acquisition at high magnification, and a novel semi-automated digital analysis pipeline implemented in QuPath, a versatile open-source digital pathology platform^41,42^ that has been employed in recent T1D studies primarily focused on immune cell analyses^22–25,43^. Our results reveal a spatially homogenous and islet size-contingent architectural organization of the endocrine pancreas; they demonstrate a notable coordination of tissue-wide pathogenic processes supported by the absence of a simple correlation between islet composition and associated immune cell burden; and they document the presence of distinct histopathological disease correlates already at the preclinical T1D stage. Accordingly, we here propose a revised natural history of T1D that readily contextualizes established hallmarks of T1D pathology with multiple novel observations.

## RESULTS

### Multiplexed IHC analysis of human pancreatic tissue sections

**Figure 1:**
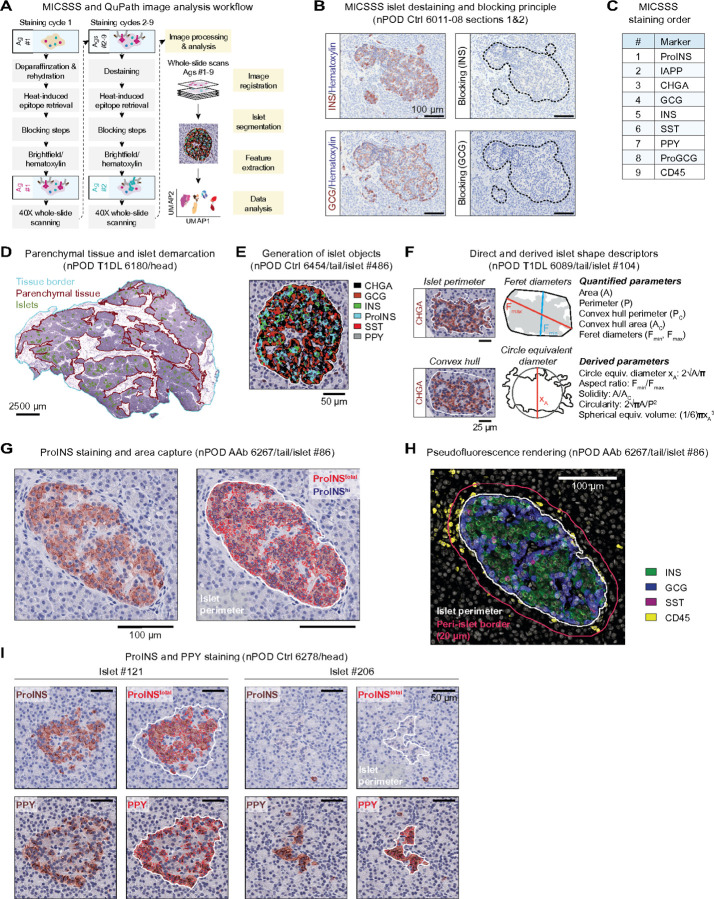
MICSSS staining and semi-automated image analysis of pancreatic tissue sections. **A.,** workflow of MICSSS staining and subsequent image analysis pipeline. In subsequent panels B.-I., donor type (Ctrl: non-diabetic, AAb: stage 1/2 T1D, T1DL: longer duration T1D), nPOD ID and pancreatic region are indicated. **B.,** MICSSS destaining/blocking illustrated for INS and GCG stains on adjacent slides. **C.,** MICSSS staining order. **D.,** demarcation of total tissue, parenchymal (exocrine/endocrine) tissue and islet areas. **E.,** generation of islet objects by additive display of six endocrine hormone stains (no PPY in present islet). **F.,** islet shape measurements and equations for derived parameters. **G.,** ProINS brightfield stain (brown), automated capture of ProINS staining areas (ProINS^total^: red traces, ProINS^hi^: blue traces), and islet perimeter (white). **H.,** pseudo-fluorescent rendering of overlaid INS, GCG, SST and CD45 stains, and demarcation of peri-islet region (border at 20mm distance from islet perimeter). **I.,** ProINS and PPY staining (brown) and respective area captures (red traces) for representative ProINS^+^PPY^+^ and ProINS^-^ PPY^+^ islets.

The detailed histopathological interrogation of healthy and diabetic human pancreatic sections remains a cornerstone of investigations into T1D pathogenesis^12^. To leverage recent advances in tissue imaging, we employed the Multiplexed Immunohistochemical Consecutive Staining on Single Slide (MICSSS) platform^44,45^, an iterative IHC assay that permits successive brightfield visualization of immunostaining patterns at high magnification (40x) across whole slides (Fig. 1A/B). Previous MICSSS applications were largely restricted to characterization of CD45^+^ hematopoietic cells^46–48^, and our adaptation for the visualization of eight pancreatic endocrine hormones in addition to CD45, in part due to larger intracellular staining areas, required adjustments to signal amplification, destaining/blocking, and an deliberate determination of staining order since our targets of interest exhibited differential sensitivity to deterioration over repeated tissue processing steps (Fig. 1C).

### Development of a semi-automated image analysis pipeline for MICSSS-stained pancreatic tissue sections

We used machine learning to analyze pancreatic whole-slide images and customized a series of specific tasks in the open-source digital pathology software QuPath^41^. We first captured total tissue specimen areas and excluded surrounding connective tissue and fat to demarcate parenchymal (exocrine and endocrine) areas (Fig. 1D). Alignment of nine immunostained images per tissue section was performed using affine transformation and achieved cell-level accuracy. Pancreatic islets, defined here as endocrine cell clusters ≥ 1,000µm^2^ (~ 10 cells/~36µm diameter), were delineated by merging the areas of six selected hormone stains with residual non-endocrine areas to create contiguous objects (Fig. 1E and Methods). Geometric and spatial properties of islet outlines were quantified to allow for a basic description of islet architecture and derivation of shape descriptors (Fig. 1F).

To delimit hormone staining areas within each islet, islet outlines were superimposed onto each image and a pixel classifier was used to segment positive staining areas. Given the complete lack of background signal and heterogeneous pattern of proinsulin (ProINS) expression as observed previously (high: Golgi/immature INS granules; low: cytoplasm/ER)^49,50^, we quantified islet areas of both high and total ProINS content (Fig. 1G). Representative images for other hormone area captures are shown in Fig.S1A. However, due to creeping introduction of some background signal over successive staining rounds, we refrained from distinguishing areas of low/high expression for all other hormones thus permitting proper demarcation of mutually exclusive staining areas (Fig. 1H). The segmented regions from each hormone stain were further used to calculate total endocrine areas (*i.e.*, union of eight hormone stains) and Jaccard indices, a statistic that quantifies the overlap of brightfield staining areas and corroborates the accuracy of our image alignment/segmentation strategy (Fig.S1B). Lastly, we incorporated the StarDist algorithm^51^ and an object classifier into our analysis pipeline to capture CD45^+^ immune cells within islets and the surrounding peri-islet areas (Figs. 1H & S1A).

The importance to include visualization of pancreatic polypeptide (PPY) expression, largely segregated to the uncinate process of the pancreatic head (PH)^52–54^, is illustrated by our observation that some PPY^+^ islets lack alpha and beta cells even in non-diabetic donors (Figs. 1I & S1C); thus, any consideration of insulin-deficient islets (IDIs) in the PH needs to include PPY analyses. Altogether, automation of multiple image analysis tasks in combination with multiplexed whole-slide image acquisition offers unique opportunities to revisit pancreatic histology in health and T1D disease *at scale*.

### Pancreatic donor cohorts and basic endocrine hormone expression patterns across T1D stages

To interrogate the histopathological evolution of T1D, we performed MICSSS staining of pancreatic tail (PT) and PH sections from four donor groups: non-diabetic controls (Ctrl), autoantibody-positive stage 1/2 T1D (AAb), short duration stage 3 T1D (T1DS; <2 years including three at-onset donors), and longer duration T1D (T1DL; 8–11 years) (Tables 1 & S1). Donor matching was performed on age and gender and, where possible, demographic (ethnicity) and clinical (body mass index/BMI) parameters; several outliers in our cohorts are described in Table S1. HLA-II risk^55–58^ was calculated to be low for Ctrl subjects and similarly elevated for AAb, T1DS and T1DL donors (Table S1 & Fig.S2A). Age, BMI, and total pancreas weight display a significant positive correlation for all donors as expected^59^. In T1DS and T1DL groups, donor age and age of T1D onset correlate near perfectly, and C-peptide and HbA1c exhibit a good fit in a one-phase exponential decay model (Fig.S2B/C).

**Figure 2:**
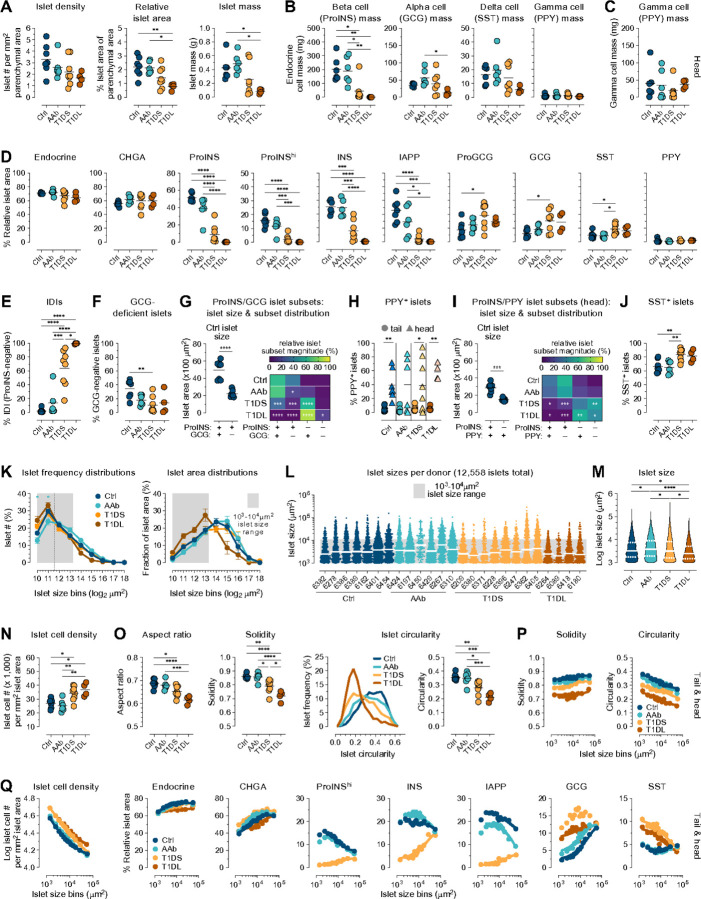
Pancreatic tissue section, islet and islet subset properties across stages of T1D progression. Unless noted otherwise, summary plots pertain to PT and display individual donor means (colored circles) and group means (black horizontal bars). **A.,** islet densities, relative areas and mass. **B./C.,** endocrine cell type mass in PT/PH was calculated from data in Fig.S2I and pancreas region weights (Table S1) excluding the two 5-year-old donors Ctrl 6382 and T1DS 6209. **D.,** fraction of indicated endocrine hormone staining areas in PT islets (“endocrine”: union of all hormone staining areas). **E.,** frequencies of IDIs (<1% ProINS content). **F.,** frequencies of GCG-deficient islets (<1% GCG content). **G.,** left: size of indicated islet subsets (individual donor medians and collective means); right: heatmap displaying relative magnitude of indicated ProINS/GCG islet subsets in each T1D stage (asterisks pertain to relative abundance of each islet subset in comparison to Ctrl donors). **H.,** frequencies of islets with ≥1% PPY content. **I.,** data display for combinatorial ProINS/PPY expression by PH islets as in panel G. **J.,** frequencies of islets with ≥1% SST content. **K.,** islet frequencies (left) and respective contributions to overall islet area (right) as a function of log_2_-transformed, bin-stratified islet sizes (data are mean±SE for individual donor groups; vertical dashed line in left panel is set at 3,000mm^2^; statistical significane between Ctrl and AAb donors therein [turquoise asterisks] was calculated by Student’s t-test for each bin). **L.,** size distribution of all 12,558 PT islets stratified according to donor group with individual donors ordered according to increasing age (white bars: medians). **M.,** islet size distributions (group median/quartiles indicated) with statistical differences calculated using a mixed model. **N.,** geometric mean of islet cell densities. **O.,** islet aspect ratio, solidity and circularity; the binned histograms display donor group-specific islet circularity distributions. **P/Q.,** properties of PT/PH islets combined from all donors in indicated groups are displayed as a function of islet sizes stratified into 14 bins; additional parameters and goodness of exponential curve fits are detailed in Fig.S4A-H. Due to notably weak CHGA and/or INS staining of PT but not PH sections from two donors (CHGA: Ctrl 6162; INS: Ctrl 6162, AAb 6450), respective data in panel D are excluded here. Unless noted otherwise, statistical analyses were conducted with ordinary one-way ANOVA and Tukey’s multiple comparisons test (*p<0.05, **p<0.01, ***p<0.001, ****p<0.0001).

Consistent with the T1D-associated loss of pancreas mass^59,60^, relative pancreas weights tend to decline in T1DS subjects, with a more pronounced reduction in our T1DL cohort (Fig.S2D). Interestingly, this decrease is also reflected in a trend toward reduction of absolute and relative parenchymal tissue section areas, and PT but not PH parenchymal areas further correlate positively with age (Fig.S2E/F). On average, we captured ~ 500 islets per tissue section, with absolute numbers broadly decreasing as T1D progresses (Fig.S2G). While islet densities show a similar 1.5–2.0-fold non-significant reduction across donor cohorts, cumulative islet areas decline significantly (Ctrl *vs.* T1DL PH: 1.8-fold; PT: 2.9-fold) corresponding to an overall 2.9–5.1-fold loss of islet mass (Figs. 2A & S2H). Quantifying cumulative islet hormone staining areas as a fraction of parenchymal tissue areas provides an initial orientation. As in earlier whole-slide imaging studies^16,17^, insulin (INS) staining areas are significantly reduced in T1DS/T1DL donors while glucagon (GCG) areas remain largely preserved (Fig.S2I). Several additional observations are noteworthy: a ~ 2.7-fold loss of chromogranin A (CHGA) from Ctrl to T1DL stage accompanies the decline of relative islet areas; total ProINS areas are larger than INS areas whereas ProINS^hi^ areas are smaller and comparable to a previous report^17^; a significant decrease of ProINS and islet amyloid polypeptide (IAPP) areas in PH but not PT of AAb donors strikingly echoes recent findings about lower beta cell volume assessed by 3D morphometric analyses^36^; somatostatin (SST) staining areas remain unaltered in all donor groups; and PPY expression, minor in PT but prominent in PH, also appears unaffected by disease progression (Fig.S2I).

For additional context, we document a progressive decline of INS:GCG expression ratios (Fig.S2J) as well as an estimation of total endocrine cell type mass. Beta cell mass wanes precipitously (Ctrl *vs.* T1DL ProINS: ~780-fold; INS: ~1,250-fold), but both alpha (ProGCG/GCG) and delta (SST) cell mass also decrease in T1DL donors, albeit to a much lesser degree (~ 3-fold; PT > PH). In contrast, gamma cell (PPY) mass is minimal in the PT (~ 1mg) yet substantial and variable in the PH (~ 20–40mg), presumably due to different uncinate process proportions in our tissue sections^52,53^ across all groups (Figs. 2B/C & S2K). These findings readily recapitulate and refine the “histopathological dynamics” of T1D progression (including loss of ~ 70–80% beta cell mass in T1DS^61^); they emphasize a profound alteration of the endocrine pancreas in the T1DL stage beyond the loss of beta cells; and they provide a general framework for our subsequent analyses focusing on ~ 25,000 individual islets captured across the four donor cohorts.

### Islet endocrine hormone contents throughout T1D progression

Extending our quantification of pancreatic endocrine contents to individual islets reveals commonalities and differences compared to cumulative staining area assessments. A profound reduction of ProINS, INS and IAPP contents in T1DS islets is similarly presaged by a trend towards decreased ProINS and IAPP but not INS expression in AAb donors; T1DS and T1DL islets present with a “compensatory increase” of ProGCG, GCG and SST expression areas reflecting the relative loss of beta cells in individual islets; and the variable PPY^+^ proportion of PH islets appears particularly large in T1DL donors (Figs. 2D & S2L). At the same time, relative endocrine and CHGA areas remain constant throughout T1D progression thus corroborating, in conjunction with the ~ 2.7-fold reduction of total CHGA staining area (S2I), a net islet loss in T1DL. Overall, differences between donor groups tend to be more pronounced in PT than PH (Figs. 2D & S2L). A direct comparison of PT and PH islets demonstrates that in all donor groups, despite comparable islet endocrine areas, PH islets express less CHGA, ProINS, INS, ProGCG, and GCG but equivalent IAPP and SST (Fig.S3A). While reduced alpha and beta cell mass in the PH of Ctrl donors has been noted earlier^53,62^, quantifying average islet hormone contents cannot resolve the potential contribution of different islet subsets to what appears an overall distinctive PH endocrine composition.

### Frequencies of islet subsets based on individual and combinatorial endocrine hormone expression

To evaluate how islet heterogeneity shapes net endocrine cell mass, we quantified islet frequencies based on their hormone content or lack thereof (defined as < 1% hormone staining area of islet area). The significant increase of IDIs (ProINS^−^ or INS^−^) with disease development (T1DS PT: ~64%, PH: 84%; T1DL PT/PH: >99%) constitutes the histopathological hallmark of T1D (Figs. 2E & S3B). An unexpected feature of the non-diabetic pancreas revealed by recent 3D mapping is the abundance of small “GCG-deficient” islets^35^. We confirm this observation in our two-dimensional interrogation by demonstrating that ~ 34% (PT) to ~ 56% (PH) of Ctrl islets are “GCG-deficient”, and that ProINS^+^GCG^−^ islets are significantly smaller than ProINS^+^GCG^+^ islets (Figs. 2F/G & S3C/D). The absence of alpha cells, as recently shown with human pseudo-islets exclusively composed of beta cells, does not appear to impinge on INS secretion dynamics^63^, suggesting that “GCG-deficient” islets constitute an integral part of endocrine pancreas physiology. Importantly, the ProINS^+^GCG^−^ islet fraction declines with T1D progression (including a significant reduction in AAb *vs.* Ctrl donor PTs), suggesting that this subset is particularly vulnerable in early T1D pathogenesis (Figs. 2G & S3D).

Furthermore, a differential abundance of PPY^+^ islets in PT *vs.* PH aligns with cumulative PPY staining patterns (Fig. 2H), and a subset stratification according to ProINS/PPY expression demonstrates that the Ctrl PH contains a ~ 10% fraction of small PPY^+^ islets lacking beta cells that increases to ~ 60% in T1DL (Fig. 2I). Delta cells are found in ~ 65% of islets, and their relative abundance is somewhat elevated in clinical T1D as noted before^64^ (Figs. 2J & S3E). Lastly, we traced the evolution of hormone co-expression patterns. For alpha cells, a modest increase of Jaccard indices for CHGA, ProGCG and GCG with T1D progression is consistent with the relative rise of ProGCG/GCG content in islets afflicted by beta cell loss. For beta cells, Jaccard indices for relevant hormone combinations slightly decline in AAb donors before markedly plunging with T1D onset reflecting the profound perturbations of INS synthesis^65^ (Fig.S3F).

### Islet architecture and its histopathological alterations

The geometric properties of islets have mostly been studied in non-diabetic pancreata^66–69^ and in accordance with these reports, Ctrl islet areas span across two orders of magnitude (~ 10^3^-10^5^µm^2^) following a frequency distribution that is notably skewed toward smaller islets as 81% (PT) to 85% (PH) of islets are found in the 10^3^-10^4^µm^2^ range; due to their smaller size, however, they respectively contribute only 44–54% to overall islet area (Figs. 2K/L & S3G/H). Although these distribution patterns are roughly maintained throughout T1D progression, disease stage-specific deviations are discernible especially in the PT where islet size positively correlates with age (Fig.S3I). A specific loss of the smallest islets (< 3,000µm^2^) in AAb *vs.* Ctrl donors (Fig. 2K) is notable since they account for ~ 50% of all islets in Ctrl subjects and their corresponding density is decreased by ~ 1.6-fold in AAb donors. Consistent with this observation, mixed model analyses demonstrate a significant increase of median islet size at the AAb stage (Fig. 2M). A subsequent decline of median islet size in T1DS and especially T1DL donors (Fig. 2K-M) is further accompanied by greater cellular densities in islets, confirming an earlier report^21^ and conceivably reflecting a local deprivation of trophic INS effects previously invoked as cause for exocrine pancreas shrinkage^60^ (Fig. 2N). Therefore, a T1D-associated islet size decrease accompanies the reduction of overall islet densities (Fig. 1A; see also ref.^70^) thus compounding the loss of islet mass. Some of these patterns are not apparent in the PH (Fig.S3G-I; in part due to the preponderance of PPY^+^ IDIs, Fig. 2I), yet higher cellular densities in T1DS/T1DL islets are comparable for both PT/PH compartments (Figs. 2N & S3J).

Recent 3D mapping of INS- and GCG-staining volumes in non-diabetic donors demonstrated that average human islet size is smaller than previously thought^35^. Working with similar assumptions about circularity and sphericity of islet objects, we used our islet area measurements to calculate median/mean islet diameters and volumes, collectively demonstrating that PT islets tend to be larger than PH islets (especially in AAb/T1DS but not T1DL donors) (Fig.S3K). Remarkably, the volume-based demonstration that islets have an average diameter of ~ 65µm^35^ closely matches our estimates that median islet diameters in Ctrl donors range from ~ 61µm (PH) to ~ 64µm (PT). Similarly, while islets with > 91µm diameter constituted ~ 26% of all islets yet contributed ~ 75% to beta cell mass in that report^35^, we find that islets above average size (> 89µm diameter) account for ~ 28% of islets (a proportion that is near identical across PT and PH as well as disease stages) and contribute ~ 70% to islet mass in all donor groups (Fig.S3L).

In contrast to the subtle alterations of islet areas and a trend towards increased islet perimeters despite declining islet sizes with advancing disease (Fig.S3M), the progression of two-dimensional shape descriptors is profound: islet aspect ratio, solidity, and circularity significantly decline from Ctrl and AAb to T1DS and T1DL stage, a pattern that again is somewhat more accentuated for PT than PH islets (Figs. 2O & S3N; aspect ratio, solidity and circularity are dimensionless measurements in the range of 0–1 that respectively describe object elongation, overall concavity, and similarity to a circular object; equations in Fig. 1F). Together, these alterations signify a stark deterioration of islet architecture. Our results indicate that inclusive whole-slide image analyses capture basic islet architectural and hormone expression features that readily align even with contemporary volumetric measurements^35,36^ and thus imbue the scope of observations reported here with greater confidence.

### A basic exponential relationship of islet size and islet properties

The heterogeneity of islet composition has long been recognized^61,71^ and in an attempt to account for disparate islet properties (including the conspicuous lack of alpha and/or beta cells specifically in smaller isles), we considered islet size as a practical organizing principle^72,73^. Correlating islet size frequency distributions in Ctrl pancreata with other islet features, we find that simple exponential relationships consistently capture these associations: increasing islet size correlates with greater islet cell numbers, solidity, endocrine/CHGA content and alpha cells but lower islet cell density, circularity as well as beta and gamma cell contents; these patterns only diverge for the smallest islets (< 2,000µm^2^) with comparatively reduced beta cell fractions, as well as for SST content which is slightly elevated in both smaller and larger islets (Figs. 2P/Q & S4A/B).

Several of these exponential relationships exhibit a striking resiliency in the face of T1D progression (endocrine and CHGA content) or are subject to a modest size-independent depression (solidity and circularity) or elevation (cellular density) indicative of pathogenetic processes encompassing islets of all sizes. While correlations of islet size with specific hormone abundance remain broadly similar in AAb *vs.* Ctrl donors, the T1DS stage features three deviations: a prominent inversion (overall reduced yet relatively higher ProINS, INS and IAPP expression by larger islets), a deterioration (ProGCG/GCG), and an emerging exponential association (SST decline as a function of islet size). In T1DL subjects, these patterns, apart from the profound beta cell loss, are mostly maintained or exacerbated (Figs. 2P/Q & S4C-H). The dynamic regulation of these exponential relationships reflects an early loss of beta cells especially in smaller islets, preservation of residual beta cell mass in larger T1DS islets as well as the broad disappearance of beta cells and markedly altered islet composition in T1DL donors (Fig.S4I). Collectively, our semi-automated whole-slide image analyses confirm central tenets of T1D pathology and readily identify multiple previously un- or under-appreciated islet properties in normal pancreata and over the course of T1D.

### Integrated histopathology throughout T1D development and progression

**Figure 3:**
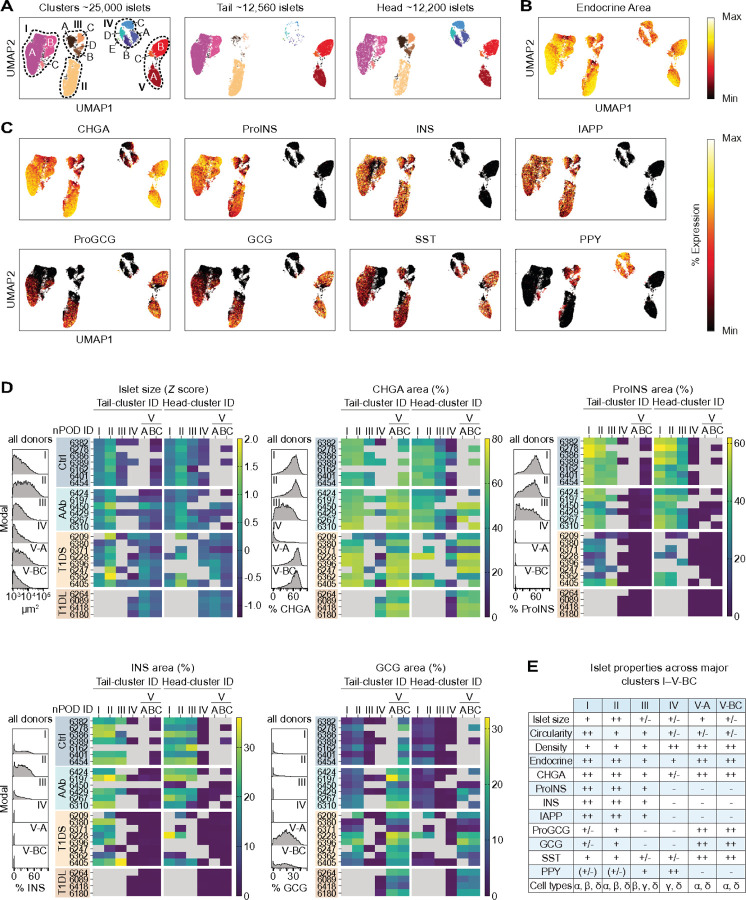
UMAP clustering of “single-islets”. **A.,** “single-islet” UMAP cluster annotation comprising all donor islets and stratification according to PT/PH regions (subcluster IV-E is sample-biased and excluded from further consideration). **B. & C.,** UMAP clustering of all islets with color gradients indicating islet size Z scores (min-max: −1.2–2.0) or relative staining area percentages for individual endocrine hormones (min-max area: endocrine and CHGA: 0–100%; ProINS: 0–90%; INS: 0–50%; IAPP: 0–40%; ProGCG: 0–60%; GCG: 0–30%; SST: 0–20%; PPY: 0–100%). **D.,** histograms pertain to indicated UMAP clusters and display islet feature distributions combined from all donor PT/PH sections; the adjacent heatmaps stratify islet size and relative hormone staining areas (CHGA, ProINS, INS, GCG) across T1D stage, individual donors (listed in order of increasing age within each group), islet cluster affiliation, and PT/PH regions. Note that not all donors have islets populating each cluster and we further omitted values if clusters contained <3 islets or <2 donors; as in Fig.2, selected CHGA (Ctrl 6162) and INS (Ctrl 6162, AAb 6450) data were also excluded (missing/excluded values rendered in gray). **E.,** summary of distinctive islet properties across UMAP clusters.

The true analytical potential of our approach to characterization of islet heterogeneity lies in elucidating complex combinatorial property patterns. We therefore adapted a dimensionality reduction tool commonly employed for transcriptomic single-cell analyses and performed UMAP clustering^74^ of all ~ 25,000 individual islets (rather than single-cells) captured in our four donor cohorts. This strategy generates five major clusters (I–V), four of which contain 3–4 subclusters (A-D), and a regional stratification illustrates that clusters III and IV are predominantly found in the PH, underscoring principal differences in the organization of endocrine compartments in PH and PT (Fig. 3A).

Visualization of individual hormone expression levels across these clusters emphasizes both shared and distinct properties of cluster-constituent islets: endocrine areas are comparable for most clusters though slightly diminished for cluster IV, a pattern that is more pronounced for CHGA expression which is strongly reduced in cluster IV and somewhat decreased in cluster III (Fig. 3B/C). Beta cell-containing islets are found exclusively in clusters I/II/III; alpha cells populate the majority but not all cluster I/II islets, are absent from cluster III and very infrequent in cluster IV, but a prominent presence in clusters V-A/V-BC; delta cells are distributed across all major clusters; and PPY^+^ islets, outside a smattering found in clusters I/II, reside in cluster III and dominate cluster IV (Fig. 3C). The islet histograms and heatmaps in Figs. 3D & S4J provide a more granular perspective on distinct cluster properties by resolving architectural and hormone expression properties at the level of individual donors and are complemented by various scatter plot arrangements to illustrate more subtle differences and allow for the clarification of significant differences (Fig.S5A-H).

Cluster stratification shows a hierarchy of average islet sizes (cluster II > > I > V-A > III ~ IV > V-BC) with near identical islet dimensions for respective PT and PH clusters (exception: AAb clusters I/II/V-BC where PT islets are bigger than PH islets) (Figs. 3D, S5A/C & S6A) indicating that the trend towards larger islets in the PT must be grounded in regionally different cluster abundances. To wit, the PH of Ctrl donors contains a prominent contingent of small cluster IV islets (median diameter: 44µm) but combined cluster I-III insulin-containing islets (ICIs) have the same median size as PT islets (64µm diameter). A similar ranking of islet circularities (cluster I > II ~ III > IV/V-A/V-BC) suggests that cluster I islets, due to their higher circularity and general preponderance in the non-diabetic pancreas (see below), may well have contributed to the historic notion of a “standard islet”^75^, and few differences in circularity or endocrine properties are observed for PT and PH islets within the same clusters (Figs. 3D, S5A/C & S6B). Also, note the differential ProINS and IAPP content for clustered ICIs (I > II > III); absence of alpha/beta cells and low CHGA but high PPY expression in cluster IV islets; and elevated ProGCG/GCG expression levels of cluster V islets in conjunction with slightly increased SST content (Figs. 3D, S4J & S5B/D). In agreement with the idea that INS may exert paracrine trophic effects, islet cell densities in clusters I/II exhibit an inverse correlation with beta cell content across disease progression while IDIs in PH cluster IV and the T1D-associated clusters V-A/BC are ~ 35% denser than cluster I/II/III ICIs (Figs. 3D & S6C). A succinct overview of these distinctive cluster properties is featured in Fig. 3E.

While UMAP analyses, by design, foreground discriminating cluster features, a comparison of islet properties within individual clusters across T1D stages also discloses some revealing differences: first, in transition from Ctrl to T1DS stage, ProINS and IAPP but less so INS content is progressively reduced in cluster I, II and III ICIs suggesting that modulation of ProINS and IAPP expression reports T1D pathogenetic processes with particular sensitivity (Figs.S5F/H). Second, opposing trajectories for islet size increase *vs.* circularity decrease in cluster II ICIs and especially cluster IV (PH) and V-A/V-BC IDIs can serve as a histopathological correlate for the dynamics of disease progression (Figs.S5E/G). Considering that larger Ctrl and AAb islets feature lesser ProINS/IAPP expression (Figs. 2Q & S4B/D), our observations are consistent with islet size-dependent disease kinetics, *i.e.* early targeting of smaller ICIs and later appearance of larger IDIs.

### Islet cluster size redistribution as an early histopathological hallmark of T1D development

**Figure 4:**
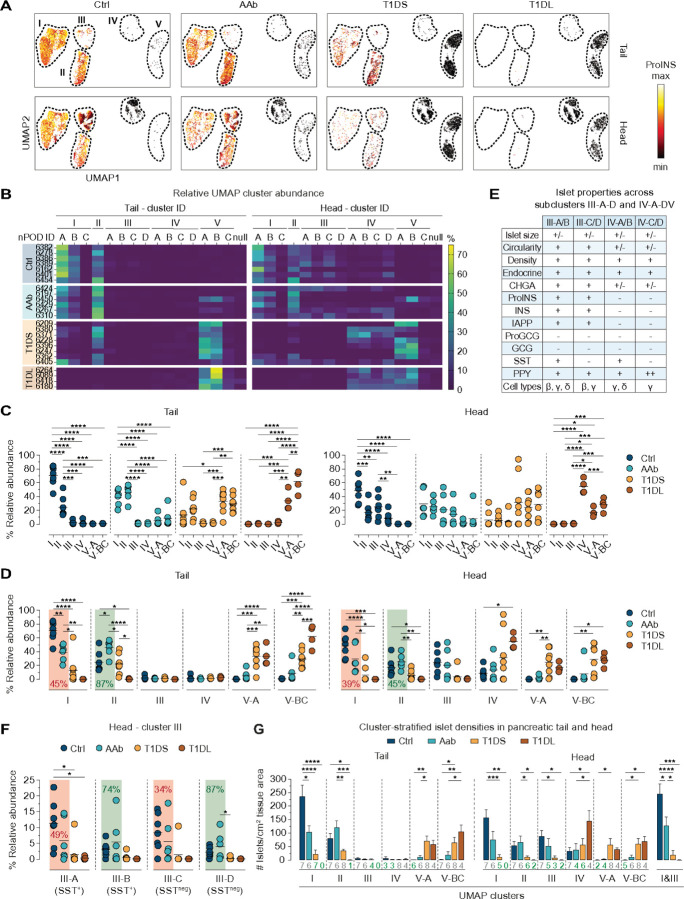
Redistribution of relative UMAP cluster sizes as a distinctive feature of early T1D development. **A.,** UMAP cluster display of all islets as a function of pancreas region and disease stage; the color gradient pertains to relative ProINS expression levels (min-max area: 0–90%). **B.,** heatmaps stratifying relative islet cluster sizes across T1D stages (individual donors listed in order of increasing age within each donor group), cluster affiliation (null, unclustered), and PT/PH regions. **C.,** relative cluster sizes in PT/PH within each donor group. **D.,**same data as in panel C now arranged for comparison of respective cluster magnitudes across T1D stages; Ctrl *vs.* AAb donors: note the relative decline of cluster I size means (red background) and accompanying increase of cluster II size means (green background). **E.,** summary of distinctive islet properties in PH subclusters III-A-D/IV-A-D (a further distinction of the four subcluster pairs according to differential immune cell burden is detailed in Fig.5F). **F.,**relative magnitudes of subclusters III-A-D in PH across T1D disease stages. **G.,** densities of cluster-stratified islets in parenchyma of PT (left) and PH (right; the far-right plot features combined cluster I/III islets since “redistribution dynamics” for these clusters are comparable, *cf.* panels D/F). Values below bars indicate the number of respective donors represented by the corresponding bar; in cases were not all donors are represented, the values are highlighted in green (data are mean±SE); for ANOVA details see Methods.

Arguably most relevant is the profound redistribution of relative cluster magnitudes with disease progression (Fig. 4A). Heatmap (Fig. 4B) and scatter plot (Fig. 4C) displays demonstrate that cluster I/II islets account for the majority of islets in Ctrl pancreata (PT: cluster I ~ 70%, II ~ 25%; PH: cluster I ~ 48%, II ~ 17%; also III ~ 25%; IV ~ 10%). In AAb donors, this distribution is markedly altered due to a 39–45% reduction of cluster I islets accompanied by a “compensatory” 45–87% increase of cluster II islets (Fig. 4D); this conclusion also applies to the individual collapse of subclusters I-A/B that are primarily distinguished by the presence/absence of delta cells (Fig.S6D), and cluster III islets in the PH including both SST^+^ and SST^−^ subsets (*i.e.*, cluster III-A/C reduction and cluster III-B/D increase) (Fig. 4E/F) thus denoting the same fate for ICIs regardless of SST content.

In transition to the AAb stage, cluster V islets emerge in some donors, and a progressive deterioration of cluster I/II magnitude in T1DS donors is accompanied by a robust increase of cluster V islets. In the PH, this pattern coincides with a relative growth of cluster IV (which “constitutively” lacks alpha/beta cells), including all of its SST^+^ and SST^−^ subclusters (Figs. 4D & S6E). The reconfiguration of cluster sizes culminates in T1DL cases where IDIs in cluster V (PT) or clusters IV/V (PH) practically eclipse a minute fraction of residual cluster II/III ICIs (Fig. 4A-F). Throughout T1D progression, distinctive differences between PT and PH cluster abundance (clusters I/II/V: PT > PH; clusters III/IV: PH > PT) remain broadly intact (Fig.S6F), indicating that despite regional differences, T1D pathogenesis proceeds in a somewhat synchronized fashion throughout the entire pancreas.

Translating the “dynamic regulation” of relative islet cluster magnitudes into the context of whole tissue sections illustrates the gradual progression of cluster-stratified islet densities with T1D stages, especially the significant reduction of AAb cluster I islet densities in the PT or of combined cluster I/III islet densities in the PH (Fig. 4G). Furthermore, the conspicuous growth of PH cluster IV with disease progression is readily explained by cluster III ICIs that, after loss of beta cells, segregate together with cluster IV IDIs, thereby raising average islet size and lowering circularity (Figs. 4E/G & S5G). Lastly, a donor-specific assessment of cluster frequency distributions (Fig. 4B) permits the identification of several outliers and associated considerations, as detailed in the legend to Fig.S6F

### Insulitis and beyond: scope and focus of the immune cell burden

**Figure 5:**
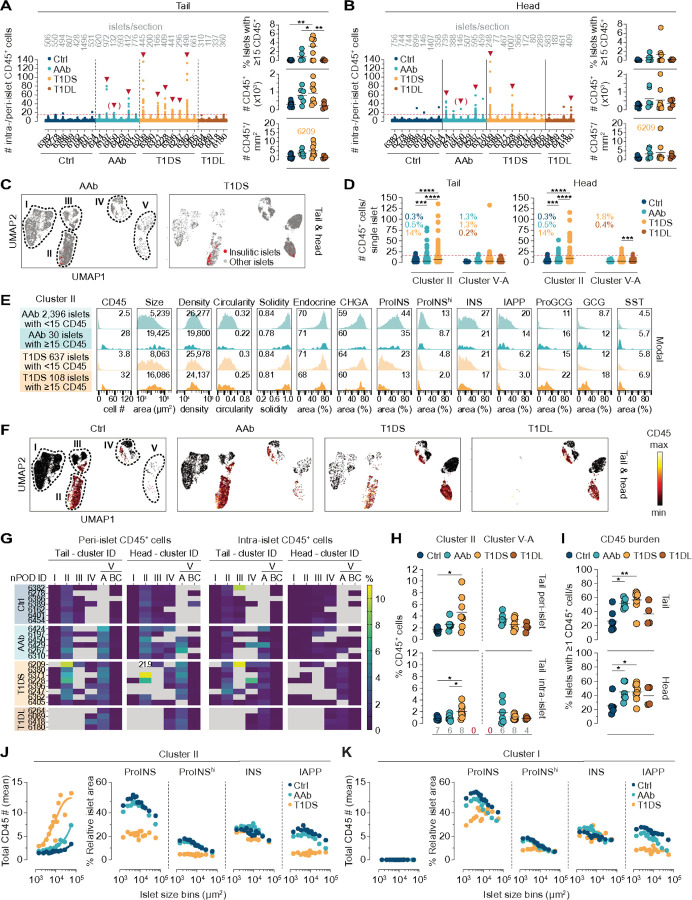
Insulitis and immune cell association with pancreatic islets. **A.,** left: total numbers of islet-associated CD45^+^ cells (*i.e.,* intra- and peri-islet) for every single PT islet from all individual donors (listed in order of increasing age within each group; gray values on top are numbers of islets captured for each donor). The dashed red line indicates the “insulitis threshold” of ≥15 islet-associated CD45^+^ cells, and red arrows highlight donors with ≥3 islets meeting the insulitis definition (AAb 6450: insulitis in two PT and one PH islet). Right: frequencies of insulitic islets (top), total number of islet-associated CD45^+^ cells in tissue sections (middle), and islet-associated CD45^+^ cell densities (CD45^+^ numbers/mm^2^ peri-islet/islet area; bottom). **B.,** data display for PH islets as in panel A. **C.,** UMAPs depicting cluster localization of insulitic islets in AAb and T1DS donors. **D.,** absolute CD45^+^ cell numbers associated with individual islets in clusters II ICIs and V-A IDIs of respective donor groups (each symbol represents an islet; dashed red line: “insulitis threshold”; color-coded values are percentages of insulitic islets in respective clusters of PT and PH). **E.,** properties of cluster II insulitic *vs.* other islets in AAb and T1DS donors (combined PT/PH islets from all AAb or T1DS donors; values are means, or medians for islet size/area; density: islet cells/mm^2^ islet area). **F.,** UMAPs of combined peri- and intra-islet CD45^+^ cell frequencies (color gradient [min-max] CD45^+^: 0–10%) across T1D stages in PT/PH. **G.,** heatmaps of average peri-/intra-islet CD45^+^ cell frequencies stratified across donor group, individual donors listed in order of increasing age within each donor group, cluster affiliation, and PT/PH regions (note out-of-range value for T1DS 6209 cluster II peri-islet CD45^+^ cell frequency; missing/excluded values in gray). **H.,** peri- and intra-islet CD45^+^ cell frequencies in PT clusters II/V-A (values above x-axis are numbers of donors analyzed for each cluster and disease stage; T1DL and Ctrl donors lack cluster II and V-A islets, respectively). **I.,** frequencies of islets with ≥1 associated CD45^+^ cell across disease stages in PT/PH. **J.,** combined PT/PH cluster II islets stratified across donor groups (Ctrl, AAb, T1DS) were “binned” according to islet size for display of associated CD45^+^ burden and ProINS/INS/IAPP expression. **K.,** same as panel J but for cluster I islets. For ANOVA details in panels A, B, D, H and I, see Methods.

To quantify immune cell abundance, distribution, and islet association, we first counted CD45^+^ immune cells localized within (intra-islet) or immediately surrounding (peri-islet) individual islets (*cf.*, Figs. 1H & S1A) to assess instances of insulitis (≥ 15 CD45^+^ cells associated with ≥ 3 islets^76^). As shown in Fig. 5A/B, automated insulitis diagnoses (0/7 Ctrl, 3/6 AAb, 6/8 T1DS, 1/4 T1DL donors) are in overall concordance with prior manual determination by nPOD pathologists (Table S1). Insulitic islets constitute an expectedly rare occurrence^12^ in our T1DS cohort (1.3% [PH] to 3.2% [PT] of islets) that nevertheless is accompanied by a ~ 2.5-fold increase of islet-associated CD45^+^ cell numbers and densities. Though not significant, it is striking that a similar immune cell burden is already present at the AAb stage regardless of even lower insulitis frequencies (0.6% PH, 0.8% PT) (Fig. 5A/B) and that its relative magnitude closely mirrors the average pancreatic increase of islet-reactive CD8^+^ T cells in AAb subjects^77^; considering specific CD8^+^ T cell frequencies in peripheral blood^77^, early immune cell recruitment to pancreatic islets may thus be mostly stochastic before enrichment of beta cell-specific CD8^+^ T cells becomes discernible at T1D onset. In the subsequent T1DL stage, islet infiltration has largely receded to levels comparable to Ctrl donors (Fig. 5A/B).

Our cluster stratification permits a refined assessment of insulitis distributions which are preferentially restricted to cluster II where 14% (PT) to 18% (PH) of islets are insulitic in T1DS cases. Such lesions are less pronounced in cluster V-A, very rare in clusters III/IV, and absent in clusters I/V-BC (Fig. 5C/D). Although insulitis frequencies are lower in AAb donors, islet-associated CD45^+^ counts in cluster II are higher than for Ctrl individuals comparing pooled donor groups, and an average of 2–3 CD45^+^ cells per islet in cluster V-A of AAb, T1DS and T1DL donors constitutes a residual presence of immune cells no longer engaging beta cells (Fig. 5D). Since cluster II islets histologically appear to constitute the principal target of autoimmune attack, we next compared the features of insulitic *vs.* non-insulitic islets in AAb and T1DS donors. Insulitic islets are noticeably larger, with a modest reduction in cellular density, circularity and solidity. Endocrine and CHGA contents remain unaltered while beta cell hormones are decreased, and alpha and delta cell hormone areas are comparatively increased (Fig. 5E). Altogether, our findings demonstrate the utility of automated image analysis strategies for expedient insulitis diagnosis, elucidation of distinctive insulitic islet properties, and capture of the overall immune cell burden that is substantial in the AAb stage despite little insulitis.

### Association of immune cells with islets: from “physiological” to preclinical to pathological

In aggregate, *i.e.*, across all disease stages and pancreas regions, ~ 52% of islet-associated immune cells are found in cluster II, ~ 8% in cluster III, ~ 13% in cluster IV, and ~ 26% in cluster V-A. Notably, ~ 40% of CD45^+^ cells therefore associate with cluster IV and V-A islets despite their lack of beta cells. Since the use of absolute islet-associated CD45^+^ cell counts in the consensus insulitis definition does not clearly account for islet size^76^, some investigators have adopted strategies for islet size correction such as quantifying immune cell numbers per mm^2^ islet area^25,28,78^. We employed a similar approach by quantifying islet-associated CD45^+^ frequencies, namely the numbers of CD45^+^ cells per nuclei in islets and peri-islet regions. Consistent with the overall cluster affiliation of islet-associated CD45^+^ cells, islets with increased CD45^+^ cell frequencies primarily localize to clusters II/V-A (in which every single islet is associated with ≥ 1 CD45^+^ cell) and, to a lesser extent, also clusters III/IV (Fig. 5F/G).

While these findings echo the distribution of insulitic islets, they further reveal a “physiological component” of immune cell infiltration since cluster II islets in Ctrl donors, accounting for ~ 17% (PH) to ~ 25% (PT) of all pancreatic islets, on average harbor ~ 1.7% CD45^+^ cells in the peri-islet region alone. This fraction increases by ~ 3-fold in T1DS donors even under exclusion of T1DS 6209, a young child with notably high peri-islet infiltrates previously considered a more aggressive “endotype 1” T1D case^79,80^. Intra-islet CD45^+^ frequencies tend to be lower but are also elevated 2.4–5.2-fold in the T1DS group, and similar considerations apply to PH cluster III islets (Figs. 5H & S6G). In contrast, cluster IV/V-A IDIs follow a different pattern: higher peri-islet CD45^+^ cell frequencies in AAb donors (PT: 3.5%, PH: 4.2%) decline to ~ 2.1% in T1DL cases with less pronounced trends also observed for intra-islet CD45^+^ cells (Figs. 5H & S6G). In fact, considering that the ratios of peri-islet to intra-islet CD45^+^ frequencies tend to rise in cluster II ICIs and fall in cluster V-A IDIs across T1D stages (Fig.S6H) may provide clues to better untangle the temporal progression of immune cell-mediated beta cell destruction. Finally, considerations of the entire spectrum of islet-associated immune cells permit the definition of an important histopathological correlate for stage 1/2 T1D: the frequency of islets associated with ≥ 1 CD45^+^ cell constitutes a straightforward metric that readily distinguishes Ctrl (25%) from both AAb and T1DS (45–55%) donors altogether underscoring the significant immune cell recruitment to islets already in the AAb stage (Fig. 5I).

### Immune cell:islet interactions and compromised islet composition in the histopathological absence of immune cells

A donor group-specific correlation between CD45^+^ burden and islet properties in cluster II, the primary histopathological locus of CD45^+^ infiltration, is confounded by the overall contingency of islet features, including CD45^+^ cell numbers and frequencies, on islet size (Figs. 2P/Q, S4A-H & S6I); instead, we compared “size-binned” cluster II islets across donor groups. Here, an increase of islet-associated CD45^+^ numbers with disease progression appears to have little bearing on islet architecture or alpha and delta cell abundance (Fig.S6J). In contrast, ProINS and IAPP, but not INS, content is markedly reduced in T1DS *vs.* Ctrl and AAb donors, especially in smaller islets (Fig. 5J). Even more striking is the observation that similar though attenuated patterns are also recorded for cluster I islets, which do not harbor any CD45^+^ cells (Figs. 5K & S6J). Cluster I islet impairment may arise following “exhaustion” of residual beta cells tasked with overwhelming metabolic demands^70,81^ or as a consequence of beta cell-targeted autoimmune attack^82^ not captured here due to its dynamic nature and/or the restricted plane of histological analysis; in all likelihood, both scenarios apply to varying degrees to different cluster I and other ICIs^6^.

Collectively, our cluster I/II islet evaluation indicates the lack of a straightforward correlation between CD45^+^ cells and islet composition, suggesting that T1D pathogenesis is at once highly dynamic (*i.e.*, CD45^+^ cells may “come and go” before complete beta cell destruction is achieved), initially targets islets below average size (compounding T1D symptomatology since smaller islets are capable of comparatively greater INS secretion^83,84^), and appears broadly synchronized since it affects the majority of islets (*i.e.*, ICIs present with reduced ProINS/IAPP expression even in the histological absence of immune cells). The utility of the insulitis concept and its proposed amendments notwithstanding^85^, considerations of the entire range of immune cell associations with pancreatic islets are therefore necessary to clarify progression of T1D autoimmune processes.

### Spatial distribution of islets throughout T1D pathogenesis

**Figure 6:**
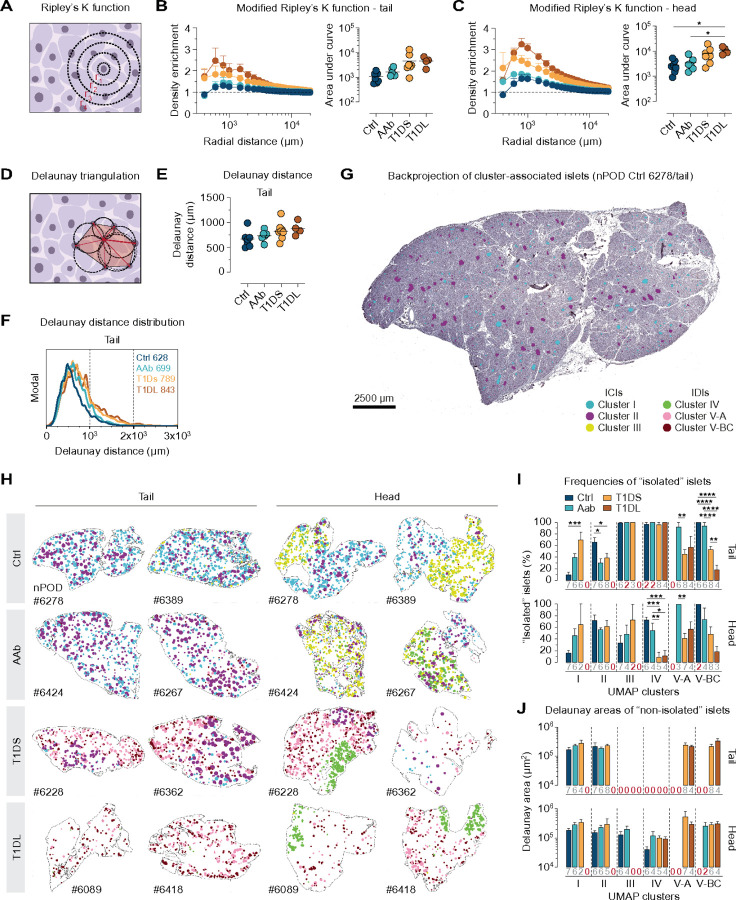
Spatial distribution of islets in the pancreas across the course of T1D pathogenesis. **A.,** Ripley’s K function: input values are radial distances between individual islets (center point) and expanding concentric circles that serve as bins to capture surrounding islets (other points) at increasing radial distances (see Methods for details). **B.,** left: islet density enrichments in PT of all groups as a function of radial islet distances ranging from 4×10^2^-2×10^4^mm; horizontal dashed line indicates random islet distribution (data are mean±SE). Right: areas under Ripley curve. **C.,** data display for PH islets as in panel B (T1DS 6380 excluded due to very scarce islets, cf. Fig.S8A). **D.,** Delaunay triangulation of a set of points/islets and associated circumcircles (gray); the center point/islet is connected by six red triangle sides to other points/islets and the average length of these lines is the mean Delaunay distance; the mean area of the six shaded triangles is the mean Delaunay area. **E.,** mean Delaunay distances between islets across T1D progression in PT. **F.,** distribution of mean Delaunay distances according to T1D stage. **G.,** *in situ* projection of islets color-coded by cluster onto representative PT section. **H.,** pancreatic tissue section outlines populated with islets in their original location color-coded according to UMAP cluster affiliation; for facilitated visual presentation, islets are rendered circular and enlarged with relative size differences preserved (color legend in panel G). **I. & J.,** Delaunay triangulation was performed separately for islets in each cluster; islets with <2 neighboring islets within an 8mm distance are “isolated islets”. I., fraction of “isolated islets”. J., mean Delaunay areas of “non-isolated” islets. The values below each bar are the numbers of donors analyzed for each cluster and disease stage (“0”: absent values or data exclusion if <3 islets or <2 donors were represented in a given cluster; instances with 2 donors only are highlighted in red to indicate need for interpretative caution; data are mean±SE). For ANOVA details in panels B, C and E, see Methods.

We employed three complementary approaches to assess the spatial distribution of islets in pancreatic tissue sections: modified Ripley’s K function, Delaunay triangulation, and direct visualization of cluster-associated islet localities. Ripley’s K function^86,87^ is a descriptive statistic for detecting deviations from spatial homogeneity, here the “aggregation” of islets in contrast to their random distribution patterns (Fig. 6A and Methods). In the PT of Ctrl donors, a modest ~ 1.2-fold density enrichment is observed for islets across median radial distances of 600–1,500µm followed by a convergence toward random distributions for greater radii (Fig. 6B). Thus, a relatively even distribution of islets throughout the PT readily aligns with recent observations obtained in 3D interrogations of non-diabetic pancreata^35^. While near identical islet distribution patterns are recorded for AAb donors, notable density enrichments occur in T1DS (~ 1.8-fold, peaking at 1,000µm) and T1DL (~ 2.6-fold, peaking at 600µm) with the right Ripley curve distribution tails still trending toward random distribution of islets, albeit less so at the T1DL stage. (Figs. 6B & S7A). Similar islet distribution patterns over the disease course are also found in the PH. However, the unique anatomy of the uncinate process (see below) is responsible for more pronounced density enrichments in all donor groups (Figs. 6C & S7A). Since islet locations are immutable, these “aggregations” reflect a progressive reduction of tissue regions with unperturbed islet densities that is driven by a relative loss of islets at shorter distances consistent with the overall decrease of islet densities, cumulative areas, and mass (Figs. 2A & S2H). Lastly, these observations are confirmed by fractal dimension analyses^88^ as illustrated in Fig.S7B.

### Progressive changes to islet neighborhoods

Delaunay triangulation organizes a set of points (islets) in a plane (tissue sections) into triangles whose circumcircles do not contain any of the points^89^; thus, mean Delaunay distances and triangle areas can serve as a more local metric for average adjacency between individual islets and their proximate neighbors (Fig. 6D). Here, Delaunay distances for PT islets increase by ~ 30% from Ctrl to T1DL stage and display a similar though blunted trajectory in the PH (Figs. 6E & S7C). At the same time, mean Delaunay distances are normally distributed in Ctrl pancreata with a slight extension of the right distribution tail representing islets that are more distant to each other; with T1D progression, the respective right distribution tails increase in particular in the PT (Fig. 6F & S7C), and further alterations can be discerned by a cluster-level evaluation of Delaunay distances as detailed in Fig.S7D-G. Collectively, our observations support the notion that the typically regionalized patterns of T1D histopathology^12^ represent residuals of a tissue-wide islet depletion, the extent of which is partially obscured by its fundamentally dispersed nature.

### Cluster-specific islet distribution patterns and the “dynamics” of T1D progression

To visualize the above relations more directly, we “projected” cluster-stratified islets back onto their original locations in the tissue sections (Figs. 6G & S7H), revealing patterns unique to specific T1D stages: in the Ctrl PT, a relatively uniform distribution of cluster I islets is accompanied by a sparser presence of cluster II islets, a balance that in AAb donors shifts to visibly fewer cluster I islets. This pattern changes more decisively in T1DS cases where the appearance of predominantly cluster V-A islets crowds out remaining cluster II islet foci, and the process is completed in T1DL with the prominent emergence of cluster V-BC islets that often appear to populate the tissue area periphery (Figs. 6H & S8A). In the PH, a similar progression pertains to the redistribution of cluster I/II/V islets, yet the most distinctive aspect is a striking regionalization of cluster III/IV islets representing the uncinate process (Figs. 6H & S8A).

We employed “islet cluster-specific” Delaunay triangulations to quantify these impressions. Here, “isolated islets” were defined as islets for which, due to fewer than two islets from the same cluster within an 8mm distance, Delaunay triangulation was not possible. As cluster I magnitudes collapse from Ctrl to T1DS stage (Fig. 4G), the fraction of “isolated islets” increases (Fig. 6I). At the same time, “isolated islet” frequencies in cluster II decline somewhat, reflecting a relative condensation of residual cluster islands that are in the process of disappearing with T1D onset (Figs. 4G & 6I). The sparse cluster III/IV islets in the PT are practically all isolated and remain so throughout all disease stages. In contrast, the more abundant cluster III ICIs in the PH become increasingly isolated as their transition to IDI status following beta cell loss leads to consolidation with cluster IV islets (*c.f.*, Fig. 4E/G); in turn, the resultant growth of cluster IV is accompanied by a steep decline of “isolated islets” therein (Fig. 6I).

“Isolated islets” in clusters V-A/BC dominate at first appearance in the Ctrl and/or AAb stage, decline in T1DS with expansion of respective cluster magnitudes (Fig. 4G), and for cluster V-BC are further reduced in T1DS (Fig. 6I). The combined “dynamics” of these changes indicate that cluster V-A represents a transitional and cluster V-BC a terminal fate. Complementary quantifications of Delaunay areas for remaining “non-isolated islets” reveal trends that partially mirror the “isolated islet dynamics” (*e.g.*, increasing Delaunay areas for cluster I islets), yet differences across disease stages remain non-significant (Fig. 6J). Altogether, these observations reinforce the notion that expanding areas of islet loss, increasingly populated by growing “diabetic” cluster V islands, encroach on progressively shrinking cluster I/II/III islands.

### Insulitis and immune cell burden in spatial context

**Figure 7:**
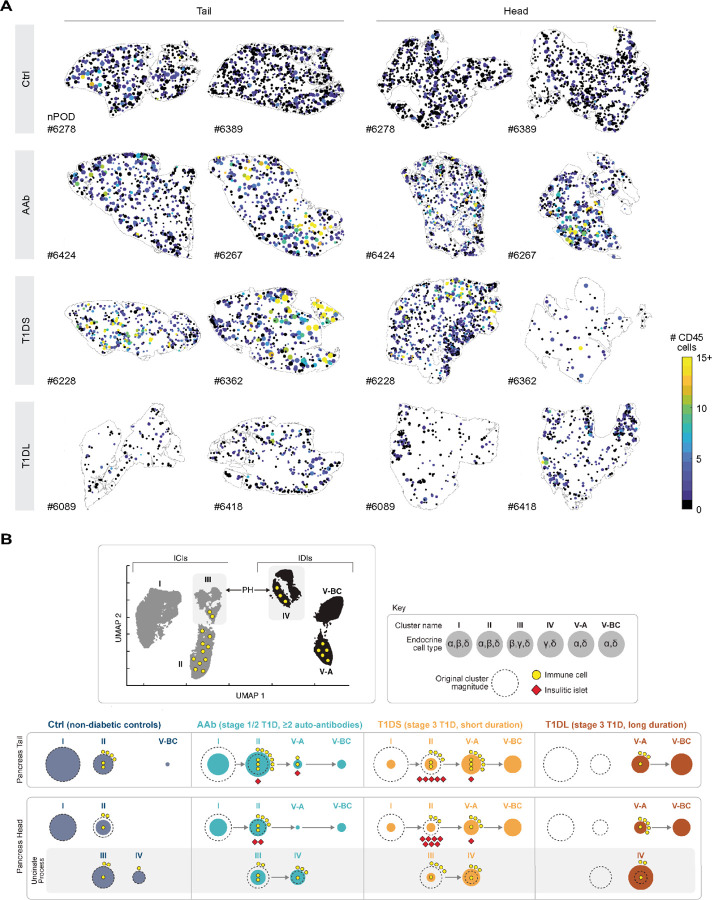
Spatial context for islet-associated immune cell burden & model summary. **A.,** pancreatic tissue section outlines (regions, T1D stage and nPOD donor IDs indicated) populated with islets color-coded according to the numbers (0–15+) of associated CD45^+^ cells (islets are rendered circular and enlarged with relative size differences preserved). **B.,** summary model: islets are allocated to UMAP clusters I – V-BC according to geometric properties, endocrine cell content, association with immune cells, and Delaunay area. Top left: major cluster properties of ICIs (gray) and IDIs (black); top right: legend. Bottom: T1D progression in the PT and PH (divided into non-uncinate and uncinate process regions). The size of colored circles represents the relative magnitude of respective islet clusters across T1D stages; their changing sizes across disease progression is indicated by arrows between adjacent circles; the dashed perimeter lines indicate the original magnitude of clusters I, II, III and IV in Ctrl donors and serve as a visual reference for cluster size changes with disease progression (a further net reduction of islet cluster magnitudes due to loss of pancreas weight is not captured here; also note that cluster III islets, upon losing beta cells, become “re-classified” as cluster IV islets).

Finally, we leveraged the “back-projection” of islet subsets to visualize the neighborhood context of insulitic lesions. Using a color gradient to display the numbers of islet-associated immune cells, the resultant images demonstrate that rare instances of insulitis in AAb and T1DS donors often are the foci of larger neighborhoods with an overall increased CD45^+^ cell burden; this pattern tends to be more pronounced for the PT than PH (Figs. 7A & S8B). And lastly, our spatial data visualization also clarifies the distinctive pathology of three previously discussed “outlier” cases and is detailed in Fig.S8A/B.

## DISCUSSION

This study combines multiplexed brightfield IHC of human pancreatic tissue sections, high-magnification whole-slide imaging, digital pathology, and development of a semi-automated image analysis pipeline implemented in the open-source pathology platform QuPath^41,42^ to trace the evolution of T1D development and progression. Integration of these modalities readily confirms the central tenets of pancreatic T1D histopathology and reveals multiple novel aspects about the dynamic constitution of the pancreas in health and T1D disease, including an essentially identical endocrine organization across pancreas regions that locates residual differences specifically to the uncinate process in the PH. Notably, we define a series of new histopathological correlates for the stage 1/2 pancreas (*i.e.*, an enhanced immune cell burden as reflected in higher frequencies of islets with ≥ 1 associated immune cell[s]; early targeting of small, including “GCG-deficient” islets; a relative reduction of total ProINS/IAPP areas; and a pronounced collapse of islet cluster I magnitude) that position AAb subjects on the cusp of developing the very histopathological hallmarks that are distinctive for T1D.

With onset of clinical T1D, several histopathological signatures emerge that collectively suggest a tissue-wide distributed rather than strictly localized disease process. Based on the proposition that islet size distribution can serve as a simple organizing principle for the substantial heterogeneity of islet composition, our observation that alterations of islet architectural features in early T1D are independent of islet size provides initial support for this notion. We further demonstrate an absence of a direct correlation between islet composition and associated immune cell abundance (*e.g.*, decreased ProINS/IAPP content even in smaller T1DS ICIs without observable CD45^+^ cells) emphasizing the importance to consider the entire spectrum of immune cell associations with islets beyond those affected by insulitis. And lastly, our analyses of spatial islet distributions foreground the “negative spaces” of T1D histopathology, namely an apparent loss of whole islets from expanding pancreas regions that is accompanied by a contraction of residual areas with “normal” islet density, thus consolidating the concept of a multifocal and more synchronized origin and progression of T1D pathology. Altogether, we therefore propose a revised natural history for T1D (Fig. 7B) that may be leveraged for more targeted investigative tasks seeking to elucidate the autoimmune pathogenesis of T1D and to advance pathology-informed considerations of interventional modalities.

In practical terms, our study offers additional analytical opportunities and exhibits several limitations. While “single-islet” UMAP clustering aids in better resolving histopathological properties and processes, our specific input variables may not be available for other studies. We therefore provide a simple key that permits an approximate “clustering” of islets based on standard three-parameter stains (INS, GCG, CD45; PPY visualization may be added to distinguish cluster III/IV islets in the PH) (Fig.S7I) and thus can be readily applied to suitable archival pancreas images. While we have excluded endocrine objects < 1,000µm^2^ for the present project, their fates are gaining increased attention^90–92^ and constitute a focus of our ongoing investigations. The refined analysis modalities and novel observations reported here are grounded in effective capture of known histopathological hallmarks of T1D progression yet will require validation in independent studies and/or larger cohorts. Our documentation of islet mass reduction in clinical T1D may be confounded by the possibility that individuals prone to T1D development have inherently smaller pancreata, a contention that at present, however, remains speculative^6^. Lastly, age-matching of pancreas donors is critical to our study design but implies different ages for disease onset; additional investigations should therefore include donors matched for age-of-onset to better account for age-dependent variables in T1D pathogenesis.

## METHODS

### Experimental specimens detail

Formalin-fixed, paraffin-embedded (FFPE) tissue sections (5mm) from pancreatic tail and head regions of organ donor pancreata were provided by the Network for Pancreatic Organ Donors with Diabetes (nPOD) and comprise 25 donors allocated to four donor groups: 7 non-diabetic control donors (Ctrl), 6 autoantibody-positive (AAb) donors, 8 donors with short duration of clinical type 1 diabetes (T1DS, <2 years), and 4 donors with longer duration of clinical T1D (T1DL, 8–11 years); donor matching across the 4 groups was performed on age and gender with additional matching for demographic (ethnicity) and clinical (body mass index/BMI) parameters where possible; further details including demographic and clinical metadata are provided in Table S1. To establish and optimize staining protocols, additional pancreatic and splenic FFPE tissue sections were provided by the nPOD consortium

### Immunohistochemistry (MICSSS)

FFPE tissue sections (5µm) were sequentially stained for eight islet hormones and CD45^+^ immune cells adjusting the multiplexed immunohistochemical consecutive staining on single slide (MICSSS) method^44,45^. Iterative staining order was empirically determined for pancreatic tissues to account for differential antigen sensitivity to deterioration during consecutive MICSSS cycles. Briefly, slides were “baked” overnight (o/n) at 37°C to ensure tissue adherence to slides in subsequent staining rounds. Following deparaffinization in histology-grade Xylene (Fisher Scientific), sections were rehydrated by immersing them in a series of graded ethanol solutions (histology-grade, Fisher Scientific) at decreasing concentrations (3×100%, 90%, 70%, and 50%) down to distilled water (5 min each) prior to heat-induced epitope retrieval (HIER) at pH6 (Citrate Buffer; ThermoFisher Scientific) in a 95°C water bath for 30 min. Tissue sections were cooled down to room temperature, endogenous hydrogen peroxidase activity was quenched by incubation with PeroxAbolish solution (Biocare Medical) for 10 min and slides were subsequently washed in Tris Buffered Saline (TBS, Cell Signaling; 2× 5 min). Non-specific background due to Fc receptor binding was blocked by incubation with DAKO serum-free protein block (Agilent) for 15 min, endogenous biotin was blocked using the DAKO Biotin-Blocking System (Agilent) according to manufacturer instructions before addition of primary antibodies listed as specified in Table S3. Target antigens were revealed after incubation with biotinylated F(ab’)_2_ donkey-raised secondary antibodies with minimal cross-reactivity (Jackson ImmunoResearch; 30 min RT), horseradish peroxidase (HRP)-conjugated streptavidin (DAKO, Agilent; 30 min), and ImmPACT AMEC Red substrate (Vector Laboratories) as per vendor’s instructions. Tissue sections were counterstained with Harris modified hematoxylin solution (Sigma), mounted with an aqueous mounting medium (DAKO Glyercgel, Agilent), whole-slide images were acquired at 40x on a NanoZoomer S60 Digital Slide Scanner (Hamamatsu) and exported as .ndpi files (for some preparatory experiments, images were acquired at 20x using a Pannoramic 250 Flash II Digital Scanner [3DHistech]; *c.f.* Fig.1B). Slides were stored protected from light at 4°C until further staining. For sequential staining of subsequent targets in MICSSS cycles #2–9, cover slips were carefully removed in hot water (∼50°C) and tissue sections were rinsed in distilled water, destained/dehydrated by immersing them in ethanol solutions at increasing concentration (50, 70, 100%; 3 min each) and xylene (3×2 min), and subsequently rehydrated through a graded ethanol series and distilled water as detailed before. Antigen retrieval at pH6 was performed for 10 min as specified above removing hematoxylin staining, endogenous and streptavidin-associated (from previous rounds of staining) hydrogen peroxidase activity, non-specific background, as well as endogenous and secondary antibody-mediated (from previous rounds of staining) biotin were blocked using PeroxAbolish, DAKO serum-free protein block, and DAKO Biotin-blocking system as above. To enable probing for target antigens using primary antibodies raised in the same species, tissue sections were incubated with 5% mouse or rabbit IgG (serum; Jackson ImmunoResearch) (depending on the primary antibody used in the immediate prior staining cycle) for 1h at RT followed by incubation with donkey-raised AffiniPure Fab Fragments (Jackson ImmunoResearch) against the previously used primary antibody species (200 µg/mL) at 4°C o/n. Subsequent primary antibody staining, target revelation, and image acquisition were conducted as described above.

### QuPath analysis pipeline

#### Image alignment.

Images were imported into QuPath version 0.2.3^41^, with each set of 9 images of a single slide bundled into an individual project. Using the CD45 image as the “base” image, the other 8 project images were each aligned to it using a custom groovy script based on QuPath’s Interactive Image Alignment function, incorporating affine transforms at 5µm resolution. A threshold pixel classifier was used to detect the entire tissue area on the CD45 image, which had been acquired last and therefore exhibited all of the accumulated tissue damage from the iterative MICSSS staining process. The pancreas tissue was then transferred to each image in the project, utilizing the pre-calculated affine transform matrix.

#### Islet detection.

Further image processing was performed with QuPath version 0.4.3 or 0.5.0. Stain separation vectors were optimized for each hormone staining round and consistently applied to every image with that antibody to spectrally unmix the AMEC chromogen from hematoxylin. CHGA was expected to mark all islets yet staining was notably faint in some PPY^+^ and small GCG^-^ islets; we therefore delineated islets with a combination of six hormone stains (CHGA, ProINS, INS, GCG, SST and PPY; ProGCG and IAPP proved redundant for this task and were therefore not included to reduce computational complexity and time). On these six stains, we segmented objects with a low-resolution pixel classifier (0.88mm), thresholding the AMEC channel to find all stained regions with an area of at least 50mm^2^. All objects were transferred to their related CD45 base image and then merged. The large resulting annotation was split into individual objects. Holes were filled in to create contiguous objects, and then all objects less than 1,000mm^2^ were removed (note that there is no official consensus about a minimal islet size that distinguishes it from smaller endocrine cell clusters; rather, our choice of 1,000mm^2^ asa suitablethreshold reflects a compromise based on historical conventions). Additionally, objects that were within 10mm of the tissue border were removed to prevent errors from incomplete islet capture and edge staining artifacts. The proto-islets were converted to detections and redistributed to all nine stains, where their overall AMEC staining intensity was measured (mean, standard deviation, min, max, and Haralick features for texture). The measurements from each object were gathered, along with shape features, and used to train a machine-learning object classifier to remove artifacts due to dust, non-specific staining, tissue damage, or loss of focus. The classifier was trained on multiple representative images from all four donor groups. We then measured and calculated shape descriptors for each final islet, and we performed Delaunay clustering on each slide with a maximum search radius of 4,000mm (1 of 24,578 islets had no neighbors within 4mm; this islet was assigned a Delaunay distance of 4mm and a Delaunay area of p x 16mm^2^).

#### Hormone staining areas & islet/endocrine cell type mass.

Islet boundaries were transferred to each of the eight hormone-stained images and a high-resolution (0.22mm) pixel thresholder was applied to find regions of positive staining. ProINS was the first stain in the series and therefore allowed for reliable differentiation of darker staining, lighter staining, and background. As CHGA is expected to be in nearly all endocrine cells, we captured all staining areas including lighter stained sections. For all other hormones, we only captured darker staining areas to avoid inclusion of background signal. The detections representing the stained area per islet were all returned to the base image and the positive area in each islet was recorded as a percentage of total islet size. For every pair of hormone stains (56 pairs), the Java JTS topography suite was used to calculate the intersection and the union of the stained regions. These areas were subsequently used to calculate Jaccard indices and relative areas of double-positive regions per islet. Additionally, we merged all hormone stains to find the total endocrine area per islet. Lastly, islet and endocrine cell type mass was calculated by multiplying relative fractions of islet areas or specific hormone staining areas with regional pancreas weights (PT or PH) (Table S1).

#### Immune cells.

Islet boundaries were expanded by 20mm to mark the peri-islet region, using a watershed algorithm to ensure that the peri-islet boundaries of neighboring islets did not overlap. Within these regions, nuclei were detected *via* the QuPath implementation of Stardist^51^, with 1mm expansion for the cytoplasm. An object classifier was trained to detect CD45^+^ immune cells. As the last stain in the MICSSS series, the CD45 image had the highest diffuse background staining within islets which furthermore varied between samples. To resolve this issue, the classifier used measurements of CD45 staining intensity in the nuclear, cytoplasmic, and membrane compartments, as well the smoothed average of cells within a 100mm neighborhood, and the difference between the cell’s intensity and that of a 10mm circular tile, including extracellular regions. After classification, the number and frequency of CD45^+^ cells inside the islet and peri-islet region were calculated.

#### CytoMAP analysis.

Islet measurements were exported to a .csv file and used for dimensionality reduction and clustering in the MATLAB implementation of CytoMAP^95^. The data input into UMAP^74^ for dimensionality reduction included: the area of eight hormone stains, the total (union) endocrine stain area, islet-associated CD45^+^ cell count, circularity, mean Delaunay area, and islet area. The total area, Delaunay area, and raw hormone areas (mm^2^) appeared log-normally distributed while the hormone staining areas as a percentage of islet areas were far from a normal distribution. Therefore, we used the log-transformed raw areas consistently. To balance the varying inputs, we calculated the z-scores for all of them. Pre-processing was performed in MATLAB. UMAP was run with the following parameters: n_neighbors = 50, min_dist = 0.1, n_epochs = 1000. Two rounds of DBScan clustering were performed on the UMAP output. First, to distinguish larger clusters, we used a high epsilon value (0.5) with a minimum number of 50 points. This yielded six clusters that largely corresponded to the visible clustering of the UMAP scatter plot (clusters 5 and 6 are in close proximity and were named V-A and V-BC). Second, to distinguish subclusters that were largely delineated by SST and GCG, we used a low epsilon (0.2) and a low minimum number of points (20). This yielded 16 clusters with some unclustered points. We used these results to divide the six major clusters (I, II, III, IV, V-A, V-BC) into subclusters for deeper analysis. The islet cluster assignment was exported from CytoMAP into a .csv file and reuploaded into QuPath as a detection measurement using custom scripts. The cluster ID was converted into a class and then class-specific Delaunay clustering with a maximum search radius of 8mm was performed.

#### Spatial analysis.

For each slide, the automatically created tissue outline was manually edited to remove peripheral areas of adipose and connective tissue and fill in regions of missing tissue to determine the pancreas parenchymal area. This was exported from QuPath as a .geojson file and then imported into MATLAB along with the islet centroid locations. To calculate spatial distribution patterns in each slide, we used a modified Ripley’s K function^96,97^ that incorporates the tissue boundary and the total pancreas density. Circles are placed at each islet with varying radii (400–10,000mm); looping through each islet, the number of other islets within that circle is counted; and the fraction of the circle area that lies inside the tissue boundary is used as a weighting factor for the number of points found. The adjusted number of points found at each distance is averaged across all islets in a slide. For Ripley analysis, this count is normalized to the number of points expected if islets were randomly distributed in the pancreas area - the average density multiplied by the area of the circle. If, at a given radius, the islets had on average the expected number of neighboring islets, the modified K value would be 1; however, if there were twice as many islets within a radius as expected, the modified K value would be 2. This allows for a comparison of spatial islet aggregation even if the pancreatic tissue sections have different sizes, islet densities, and complexities. These data were further used for fractal spatial analysis following the method of Jo *et al.*^88^. For each slide, search radii and boundary-adjusted islet counts were plotted on a log-log graph and the slope of the best-fit line was calculated, excluding radii ≤600µm; the slopes, or fractal dimensions, of each slide were compared to demonstrate average changes in islet distribution with disease progression.

#### Data visualization.

To ease data visualization and figure creation, the large matrix of islet measurements, including single and double hormone areas, locations, shape descriptors, spatial measurements and UMAP clusters were converted to .fcs files for visualization in Flowjo 10.10.0 (BDBiosciences) using the writeFCS function^98^. In some cases, contrast and brightness were adjusted for entire brightfield images using Adobe Photoshop, and scale bars were added in Fiji/imageJ. Pseudofluorescence images were generated in QuPath using the spectrally separated AMEC channels per stain and the previously calculated affine transforms.

### HLA haplotype T1D risk classification

HLA class I and class II haplotype data were classified for T1D risk according to published data and are summarized in Table S1. HLA class II DR-DQ genotypes were binned based on T1D risk, high, moderate, neutral or protective, determined from individuals of European ancestry^55^. Bins were assigned numerical values for the purposes of this study, high risk (+2), moderate risk (+1), neutral (0), or protective (−1). Alternatively, HLA class II risk was classified using published odds ratios (OR) calculated for individuals of European (DR-DQ haplotype combinations^56^), African (individual DR-DQ haplotypes^57,58^), or admixed Hispanic/Latino descent (individual DR-DQ haplotypes^58^). HLA class I risk was classified for HLA-A and - B genotypes using OR calculated from individuals of European descent^99^.

### Quantification and statistical analysis

Data analysis and graphical representation were performed in GraphPad Prism 9 and 10 (GraphPad Software). Normal distribution was determined by D’Agostino-Pearson test. Statistical significance was assessed by unpaired or paired Student’s ttests or non-parametric Mann-Whitney U test as applicable for comparison between two groups; by one-way ANOVA with Tukey’s multiple comparisons post hoc testing for analysis of more than two groups with normally distributed values; or by one sample t test using a hypothetical mean as indicated. Analyses of islet sizes were conducted using mixed effect models with subjects as random effects specifying a compound-symmetry correlation matrix in the model, and orthogonal contrasts were used for pair-wise comparison of the four donor groups. Summary data are displayed as scatter plots with mean, violin plots with median and quartiles, bar diagrams (mean±SE), frequency distributions (mean or median with interquartile range as indicated), or correlation plots adopting the following convention: *p<0.05, **p<0.01, ***p <0.001, ****p<0.0001; ns or no symbol, non-significant.

Missing and excluded data: for the purpose of the present study, pancreatic islets are defined as endocrine objects ≥1,000mm^2^ (~10 cells, ~36mm diameter); accordingly, we excluded smaller endocrine structures and single cells from all of our analyses. Information about HLA haplotypes and HbA1c values was available for most but not all donors (Table S1 & Fig.S2A/C). Subcluster IV-E (Fig.3A) is sample-biased and was not further considered. In some analyses of UMAP cluster-stratified islets, not all donors have islets present in all clusters; in addition, we excluded values if <3 islets or <2 donors were represented in a cluster. The resultant numbers of donors analyzed for each cluster are featured on top of respective x-axes in Figs.6I/J, S5 & S6A-C/G/H with absent values indicated by “0” in red font and the presence of only two donors also highlighted in red font to indicate the need for interpretative caution. CHGA and INS staining of Ctrl 6162 PT tissue sections and INS staining of AAb 6450 PT sections was notably weak; in the absence of CHGA and INS staining irregularities in the corresponding PH sections as well as normal ProINS and IAPP staining in both PT and PH sections, we attribute this observation to a technical staining issue and therefore excluded the respective PT CHGA and INS data from Figs.2D, 3D, S1C, S2I-K, S3A/B/F & S5B/F. (Figs.2B/C & S2K), and PH data from T1DS 6380 was excluded from modified Ripley’s K analyses due to very scarce islets (Fig.6C & S8A).

## Supplementary Material

1
