# Supplementary material for: Integrated histopathology of the human pancreas throughout stages of type 1 diabetes progression
Source: Res Sq. 2025 Jun 10:rs.3.rs-6673858. Preprint. [Version 1] doi: 10.21203/rs.3.rs-6673858/v1 (PMC12204496; doi:10.21203/rs.3.rs-6673858/v1)
Supplement: 1 [file NIHPPrs6673858v1-supplement-1.pdf]

Supplementary Files

This is a list of supplementary files associated with this preprint. Click to download.

- Table120250510.pdf
- SupplementalFiguresS1S820250510.pdf
- SupplementalTableS120250510.xlsx
- SupplementalTableS320250510.pdf
